# Simpson's paradox and the impact of donor-recipient race-matching on outcomes post living or deceased donor kidney transplantation in the United States

**DOI:** 10.3389/fsurg.2022.1050416

**Published:** 2023-01-09

**Authors:** Kaikai Lv, Yangyang Wu, Wenhui Lai, Xiaowei Hao, Xinze Xia, Shuai Huang, Zhenjun Luo, Chao Lv, Yuan Qing, Tao Song

**Affiliations:** ^1^ Department of Urology, The Third Medical Centre, Chinese People’s Liberation Army (PLA) General Hospital, Beijing, China; ^2^ Medical School of Chinese People’s Liberation Army (PLA), Beijing, China; ^3^ Department of Postgraduate, Hebei North University, Zhangjiakou, China; ^4^ Department of Urology, Shanxi Medical University, Taiyuan, China; ^5^ Affililated Hospital of Weifang Medical University, School of Clinical Medicine, Weifang Medical University, Weifang, China

**Keywords:** kidney transplantation, race-matching, Simpson’s paradox, patient survival, graft survival

## Abstract

**Background:**

Race is a prognostic indicator in kidney transplant (KT). However, the effect of donor-recipient race-matching on survival after KT remains unclear.

**Methods:**

Using the United Network for Organ Sharing (UNOS) database, a retrospective study was conducted on 244,037 adults who received first-time, kidney-alone transplantation between 2000 and 2019. All patients were categorized into two groups according to donor-recipient race-matching, and the living and deceased donor KT (LDKT and DDKT) were analyzed in subgroups.

**Results:**

Of the 244,037 patients, 149,600 (61%) were race-matched, including 107,351 (87%) Caucasian, 20,741 (31%) African Americans, 17,927 (47%) Hispanics, and 3,581 (25%) Asians. Compared with race-unmatching, race-matching showed a reduced risk of overall mortality and graft loss (unadjusted hazard ratio (HR) 0.86, 95% confidence interval (CI) 0.84–0.87; and unadjusted HR 0.79, 95% CI: 0.78–0.80, respectively). After propensity score-matching, donor-recipient race-matching was associated with a decreased risk of overall graft loss (*P* < 0.001) but not mortality. In subgroup analysis, race-matching was associated with higher crude mortality (HR 1.12, 95% CI: 1.06–1.20 in LDKT and HR 1.11, 95% CI: 1.09–1.14 in DDKT). However, race-matching was associated with a decreased risk of graft loss in DDKT (unadjusted HR 0.97, 95% CI: 0.96–0.99), but not in LDKT. After propensity score-matching, race-matching had better outcomes for LDKT (patient survival, *P* = 0.047; graft survival, *P* < 0.001; and death-censored graft survival, *P* < 0.001) and DDKT (death-censored graft survival, *P* = 0.018). Nonetheless, race-matching was associated with an increased adjusted mortality rate in the DDKT group (*P* < 0.001).

**Conclusion:**

Race-matching provided modest survival advantages after KT but was not enough to influence organ offers. Cofounding factors at baseline led to a contorted crude conclusion in subgroups, which was reversed again to normal trends in the combined analysis due to Simpson's paradox caused by the LDKT/DDKT ratio.

## Introduction

For most patients with end-stage renal disease (ESRD), kidney transplantation (KT) is the preferred treatment because it has a superior survival to dialysis (1). In the United States, KT has shown an annual increasing trend. Additionally, a large body of literature (2–6) has focused on the effect of donor and/or recipient race on post-transplant outcomes, albeit with inconsistent results. Donor-recipient race-matching is considered a potential prognostic factor for organ transplant outcomes, since receiving organs from the same race increases the possibility of genetic and physiological similarities between donors and recipients and thereby reduces the possibility of rejection events (7). However, few studies (8–12) have investigated the impact of donor-recipient race-matching on post-transplant survival, especially in KT. Although some studies have noted reduced survival of patients or grafts in race-unmatched organ transplantation, conflicting results have been reported in several studies. In addition, most prior studies are now outdated, and the different population classifications affect comparability among different studies. Understanding the impact of race-matching on patient and graft survival is crucial for improving KT outcomes.

Therefore, we performed the present study to evaluate the potential implication of donor-recipient race-matching on post-KT patient and graft survival utilizing United Network for Organ Sharing (UNOS) database. We hypothesized that donor-recipient race-matching in KT would improve patient and graft survival compared with unmatching, due to genetic and physiological similarities.

## Methods and materials

### Data source and study design

We obtained the data from the UNOS database. Our institutional review board (IRB) deemed this study exempt from IRB approval because no patients or center identifiers were included in this analysis. We retrospectively examined adults (≥18 years) who received a first-time, kidney-alone transplant from January 1, 2000 to December 31, 2019. The cohort was classified into race-matched and unmatched groups according to whether the donors and recipients were of the same ethnicity. In addition, we performed subgroup analyses by donor type (living and deceased). Specific races (Caucasian, African American, Hispanic, and Asian) were investigated, and other races (including American Indian, Pacific Islander, and unknown races) were excluded because of small sample sizes.

### Variables examined and outcome measures

The present study included recipient, donor, and transplant period variables. The primary covariates of interest were the races of recipients and donors. Demographic factors of recipient and donor included age, body mass index (BMI), gender and race. The primary causes of ESRD were classified into five groups [glomerular disease, diabetes mellitus (DM), hypertension, and polycystic kidneys and other]. We also checked other recipient information, such as insurance type, education level, dialysis before transplant, and days on the waitlist. Donor type and cause of death for deceased donors were also examined. Finally, transplant factors were also evaluated as covariables, including transplant time periods, human leukocyte antigen (HLA) mismatch, delayed graft function (DGF), cold ischemic time, and reported any acute rejection.

The endpoints were crude and were adjusted for patient and graft survival, and death-censored graft survival in both LDKT and DDKT.

### Statistical analysis

Comparison of clinical and demographic characteristics between the race-matched and unmatched groups was performed using Student's *t*-test (for continuous variable) and *χ*^2^ test (for categorical variable). We censored recipients who lost to follow-up prior to death. The cumulative survival rate was evaluated with Kaplan–Meier curves, and log-rank tests were used to determine survival differences. Survival analysis was conducted with Cox proportion hazard ratio regression models for the overall data and for subgroups to determine the magnitude of difference. Considering statistically significant differences in clinical factors between the race-matched and unmatched cohorts, we used propensity score-matching for 1 : 1 matching of race-matched and unmatched patients. Data missing patients were exclude from the matching model. In the subgroup analysis, propensity scores were calculated using recipient covariates (age, BMI, gender, race, causes of ESRD, primary insurance, education level, and dialysis before KT) and donor covariates (age, BMI, gender, and race), as well as organ-specific factors (HLA mismatch, DGF, cold ischemic time, and acute rejection), but with the addition of donor type as a covariate in the overall analysis. In addition, a balance diagnosis was performed by comparing the baseline characteristics between race-matching and race-unmatching groups (Supplementary Figure S1).

For all analyses, all *P*-values were 2-sided, and statistical significance was set at *P* < 0.05. Means are presented with standard deviations (SD), and hazard ratios (HR) are presented with 95% confidence intervals (CI). All analyses were conducted using R (version 3.6.2) within RStudio (version 1.1.456).

## Results

### Cohort statistics

From January 1, 2000 to December 31, 2019, 342,990 patients undergoing KT were identified in the United States according to UNOS. Excluding previous transplants (*n* = 35,630), children (*n* = 35,351), multiple organ transplantation (9,490), dual KT (*n* = 5,102), ABO incompatibility (*n* = 2,276), foreign donors (*n* = 7), and minorities (*n* = 11,097), the final study subjects of 244,037 were enrolled in the study. The mean age of donors and recipients was 40.21 ± 14.60 years and 51.68 ± 13.38 years with percentages of male being 52.89% (*n* = 129,072) and 61.06% (*n* = 149,008), respectively.

**Figure 1:**
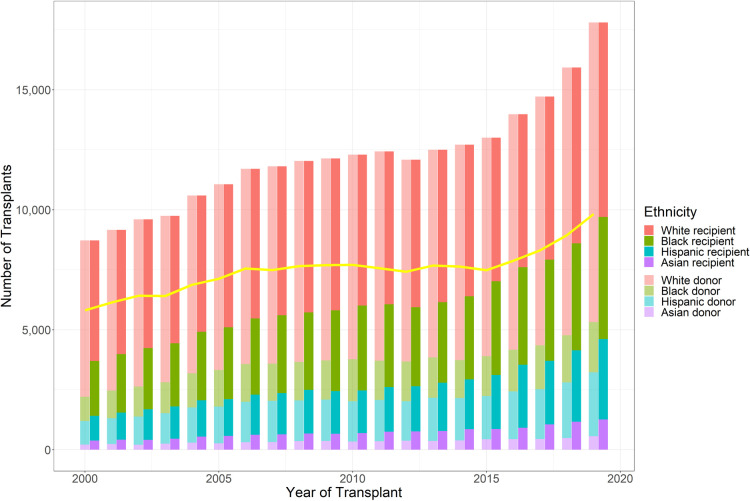
Number of kidney transplants performed during the study period, stratified by donor and recipient race (Caucasian, African American, Hispanic, and Asian). Line shows number of race-matched kidney transplants performed yearly (based on the UNOS database).

The race distribution of recipients was as follows: Caucasian (*n* = 123,646, 50.67%), African American (*n* = 67,610, 27.70%), Hispanic (*n* = 38,280, 15.69%), and Asian (*n* = 14,501, 5.94%). The racial distribution of donors was as follows: Caucasian (*n* = 171,632, 70.33%), African American (*n* = 31,589, 12.94%), Hispanic (*n* = 33,789, 13.85%), and Asian (*n* = 7,027, 2.88%) (Figure 1).

Overall, 149,600 (61.30%) patients received kidneys from race-matched donors, whereas 94,437 (38.70%) patients received kidneys from race-unmatched donors. A total of 107,351 (86.82%) Caucasians, 20,741 (30.68%) African Americans, 17,927 (46.83%) Hispanics, and 3,581 (24.69%) Asians received race-matched kidneys (Table 1). The percentage of annual donor-recipient race-matching varied from 55.16% to 66.99%, with a decreasing trend over time. The number of KT cases ranged from 8,722 to 17,809 during the 20-year study period, with an upward trend over time, particular after 2015 (Figure 1).

**Table 1:** Baseline characteristics stratified by donor-recipient race-matching.

	Race-matched (*n *= 149,600)	Race-unmatched (*n *= 94,437)	*P*-value
**Recipient age, *n* (%)**			
≥60	48,545 (32.4)	29,775 (31.5)	<0.001
Mean, (±SD)	51.59 (±13.71)	51.81 (±12.85)	<0.001
**Recipient BMI, mean (±SD)**	28.00 (±5.41)	28.08 (±5.42)	0.001
**Recipient gender, male, *n* (%)**	91,845 (61.4)	57,163 (60.5)	<0.001
**Recipient ethnicity, *n* (%)**			<0.001
Caucasian	107,351 (71.8)	16,295 (17.3)	
African America	20,741 (13.9)	46,869 (49.6)	
Hispanic	17,927 (12.0)	20,353 (21.6)	
Asian	3,581 (2.4)	10,920 (11.6)	
**Cause of ESRD, *n* (%)**			<0.001
Glomerular disease	30,888 (20.6)	15,593 (16.5)	
DM	38,889 (26.0)	28,428 (30.1)	
Hypertension	30,120 (20.1)	29,862 (31.6)	
Polycystic kidneys	18,824 (12.6)	6,015 (6.4)	
Other	30,879 (20.6)	14,539 (15.4)	
**Recipient insurance, *n* (%)**			<0.001
Public	84,688 (56.6)	71,121 (75.3)	
Private	64,902 (43.4)	23,311 (24.7)	
**Recipient education level, *n* (%)**			<0.001
College or graduate degree	74,182 (49.6)	40,283 (42.7)	
Pre-college	61,520 (41.1)	45,045 (47.7)	
**Dialysis before KT, years, *n* (%)**			<0.001
No	33,939 (22.7)	8,022 (8.5)	
Yes	114,685 (76.7)	85,952 (91.0)	
**Days on waitlist, mean (±SD)**	569.25 (±615.47)	909.24 (±784.06)	<0.001
**Donor age, *n* (%)**			
≥60	12,520 (8.4)	8,226 (8.7)	0.003
Mean (±SD)	40.63 (14.06)	39.55 (15.40)	<0.001
**Donor BMI, mean (±SD)**	27.22 (±5.73)	27.41 (±6.54)	<0.001
**Donor gender, male, *n* (%)**	74,119 (49.5)	54,953 (58.2)	<0.001
**Donor ethnicity, *n* (%)**			<0.001
Caucasian	107,351 (71.8)	64,281 (68.1)	
African American	20,741 (13.9)	10,848 (11.5)	
Hispanic	17,927 (12.0)	15,862 (16.8)	
Asian	3,581 (2.4)	3,446 (3.6)	
**Donor type, *n* (%)**			<0.001
Living donor	73,395 (49.1)	9,199 (9.7)	
Deceased donor	76,205 (50.9)	85,238 (90.3)	
**Deceased donor cause of death, *n* (%)**			<0.001
Anoxia	19,897 (13.3)	24,819 (26.3)	
Cerebrovascular/Stroke	25,887 (17.3)	28,076 (29.7)	
Head trauma	28,081 (18.8)	29,613 (31.4)	
Other	2,340 (1.6)	2,730 (2.9)	
**Period of transplantation, *n* (%)**			<0.001
2000–2004	31,651 (21.2)	16,173 (17.1)	
2005–2009	37,517 (25.1)	21,236 (22.5)	
2010–2014	38,004 (25.4)	24,022 (25.4)	
2015–2019	42,428 (28.4)	33,006 (35.0)	
**HLA mismatch, *n* (%)**			<0.001
<3	37,558 (25.1)	310 (7.7)	
≥3	111,466 (74.5)	87,011 (92.1)	
**Cold ischemic time, ≥12 h, *n* (%)**	55,176 (36.9)	62,431 (66.1)	<0.001
**Delayed graft function, *n* (%)**	20,193 (13.5)	25,782 (27.3)	<0.001
**Acute rejection, *n* (%)**	12,318 (8.2)	9,214 (9.8)	<0.001

BMI, body mass index; ESRD, end-stage renal disease; DM, diabetes mellitus; KT, kidney transplant; HLA, human leukocyte antigen; SD, standard deviation.

### Baseline characteristics

Differences in baseline characteristics were observed between race-matched (*n* = 149,600) and race-unmatched groups (*n* = 94,437). Specifically, recipients who received allografts from race-matched donors were younger (51.59 ± 13.71 vs. 51.81 ± 12.85, *P* < 0.001), presented with a lower BMI (28.00 ± 5.41 vs. 28.08 ± 5.42, *P* = 0.001), tended to be white (71.8%) and male (61.4% vs. 60.5%, *P* < 0.001), were less likely to have public insurance (56.6%) and dialysis prior to KT (76.7%), were more likely to get college or graduate degrees (49.6%), and spent an average of 340 days shorter on the waitlist than those receiving allografts from race-unmatched donors. The cause of ESRD was more likely to be DM (*n* = 38,889, 26.0%) in race-matched group, while it was to be hypertension (*n* = 29,862, 31.6%) in race-unmatched group.

Race-matched donors were older (40.63 ± 14.06 vs. 39.55 ± 15.40, *P* < 0.001), had a lower BMI (27.22 ± 5.73 vs. 27.41 ± 6.54, *P* < 0.001), tended to be female (50.5%), and were more likely to be living donors. In both groups, the highest racial proportion amongst donors was Caucasian (Table 1).

The number of transplant recipients showed an upward trend in both groups, with the highest number observed between 2015 and 2019. There were statistically significant differences between race-matched and unmatched-groups in terms of HLA mismatch (≥3), cold ischemic time (≥12 h), DGF, and acute rejection (Table 1).

### Patient and graft survival

**Figure 2:**
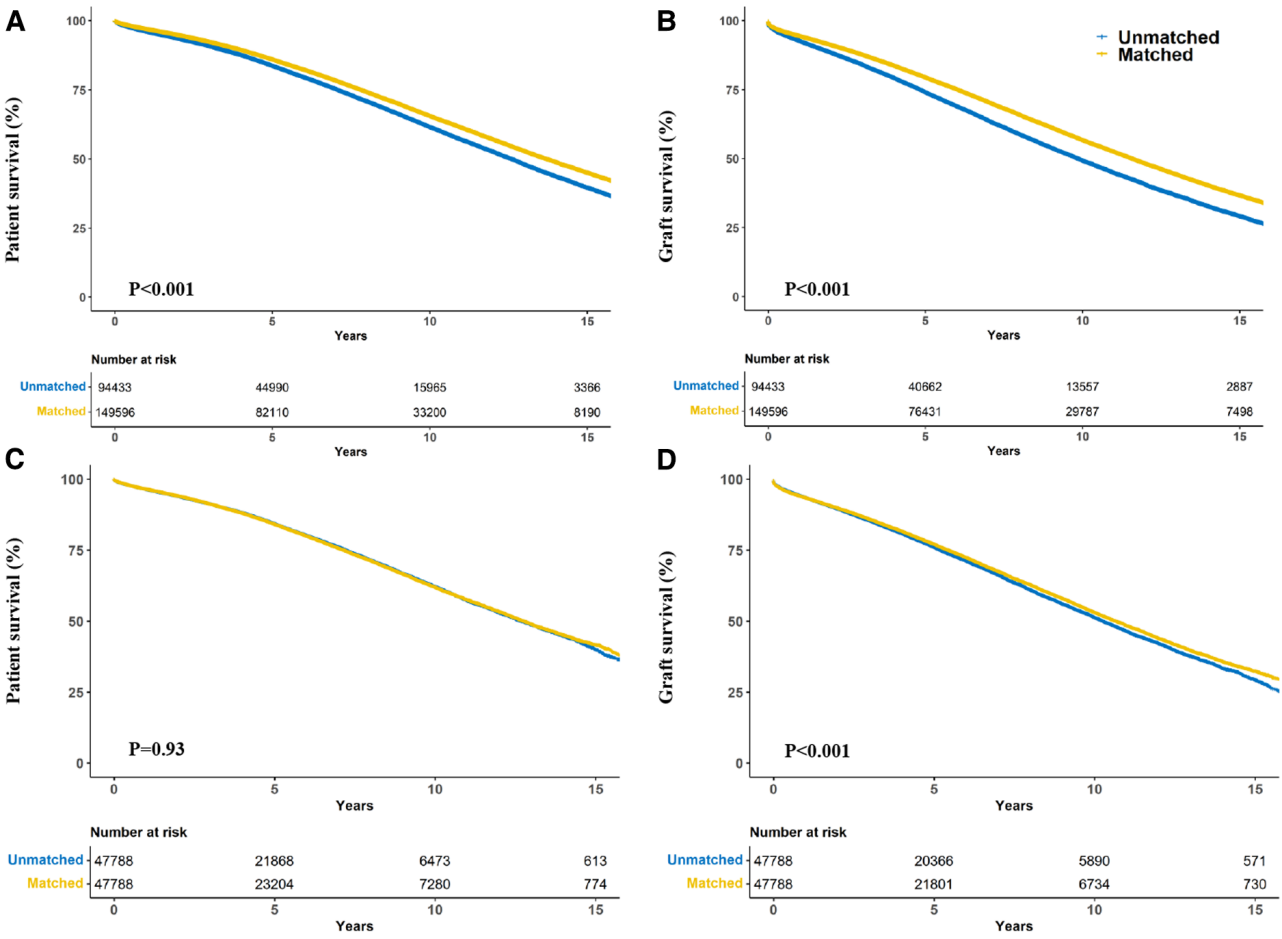
Crude kaplan–meier (**A,B**) and propensity score matching kaplan–meier (**C,D**) curves estimating patient (**A,C**) and graft (**B,D**) survival for kidney transplant recipients stratified according to donor-recipient race-matching vs. unmatching.

Race-matched recipients survived longer than unmatched recipients (*P* < 0.001) (Figure 2A) with similar result observed for graft survival (*P* < 0.001) (Figure 2B). Compared with race-unmatched patients, race-matched patients experienced a 14% and 21% reduction in the risk of unadjusted mortality and graft failure, respectively (HR 0.86, 95% CI: 0.84–0.87, *P* < 0.001 and HR 0.79, 95% CI: 0.78–0.80, *P* < 0.001) (Table 2). The cumulative 3-, 5-, and 10-year overall survival rates in patients receiving race-matched kidneys were 1.5%, 2.33%, and 4.02% respectively, higher than those in patients who received race-unmatched kidneys, whereas there was no significant difference in 1-year overall patient survival rates between race-matched and race-unmatched groups. Moreover, when examining patient survival at 1-, 3-, 5-, and 10-year within individual races, race-matched patients demonstrated a significant improvement compared to race-unmatched patients (Table 3). Race-matched patients demonstrated a significant improvement in individual-race and overall graft survival compared to race-unmatched patients (Table 4).

**Table 2:** Univariate cox regression model for mortality and graft loss.

	Mortality	*P*-value	Graft loss	*P*-value
Overall race-matched	0.86 (0.84–0.87)	<0.001	0.79 (0.78–0.80)	<0.001
LDKT race-matched	1.12 (1.06–1.20)	<0.001	1.01 (0.97–1.07)	0.565
DDKT race-matched	1.11 (1.09–1.14)	<0.001	0.97 (0.96–0.99)	0.001

LDKT, living donor kidney transplantation; DDKT, deceased donor kidney transplantation.

**Table 3:** Effect of race-matching on unadjusted kaplan–meier estimates of patient survival overall and stratified by race.

	1-year survival	3-year survival	5-year survival	10-year survival
Overall				
Matched	97.06% (96.97–97.15)	92.42% (92.28–92.56)	86.10% (85.90–86.29)	65.65% (65.32–65.99)
Unmatched	96.16% (96.04–96.28)	90.92% (90.72–91.11)	83.77% (83.50–84.04)	61.63% (61.17–62.09)
*P*-value	0.23	<0.001	<0.001	<0.001
Caucasian				
Matched	96.86% (96.75–96.96)	91.90% (91.73–92.08)	85.11% (84.87–85.35)	63.52% (63.13–63.92)
Unmatched	95.41% (95.09–95.74)	89.17% (88.67–89.68)	80.39% (79.71–81.09)	57.26% (56.21–58.33)
*P*-value	<0.001	<0.001	<0.001	<0.001
African American				
Matched	96.93% (96.69–97.16)	91.99% (91.60–92.38)	85.83% (85.30–86.37)	65.66% (64.76–66.57)
Unmatched	96.09% (95.91–96.27)	90.48% (90.19–90.76)	83.21% (82.82–83.60)	59.92% (59.26–60.59)
*P*-value	<0.001	<0.001	<0.001	<0.001
Hispanic				
Matched	98.15% (97.95–98.35)	95.33% (95.00–95.66)	91.17% (90.69–91.65)	77.08% (76.17–77.99)
Unmatched	96.45% (96.19–96.71)	92.12% (91.73–92.52)	85.57% (85.00–86.14)	64.44% (63.41–65.48)
*P*-value	<0.001	<0.001	<0.001	<0.001
Asian				
Matched	98.50% (98.10–98.90)	96.35% (95.70–96.99)	93.59% (92.69–94.50)	81.98% (80.16–83.84)
Unmatched	97.05% (96.72–97.37)	93.31% (92.81–93.82)	88.33% (87.63–89.04)	71.67% (70.41–72.96)
*P*-value	<0.001	<0.001	<0.001	<0.001

**Table 4:** Effect of race-matching on unadjusted kaplan–meier estimates of graft survival overall and stratified by race.

	1-year survival	3-year survival	5-year survival	10-year survival
Overall				
Matched	94.53% (94.41–94.64)	87.74% (87.56–87.91)	79.58% (79.35–79.81)	56.86% (56.52–57.21)
Unmatched	92.74% (92.58–92.91)	84.11% (83.86–84.36)	74.16% (73.84–74.48)	49.37% (48.91–49.84)
*P*-value	<0.001	<0.001	<0.001	<0.001
Caucasian				
Matched	94.43% (94.29–94.57)	87.94% (87.73–88.14)	79.81% (79.54–80.08)	56.72% (56.33–57.13)
Unmatched	92.07% (91.66–92.49)	83.53% (82.94–84.13)	73.51% (72.75–74.27)	49.98% (48.94–51.05)
*P*-value	<0.001	<0.001	<0.001	<0.001
African American				
Matched	93.00% (92.66–93.54)	82.77% (82.23–83.31)	72.42% (71.74–73.09)	48.41% (47.51–49.33)
Unmatched	91.98% (91.73–92.23)	81.79% (81.43–82.16)	70.74% (70.27–71.21)	44.38% (43.74–45.02)
*P*-value	<0.001	<0.001	<0.001	<0.001
Hispanic				
Matched	96.32% (96.04–96.60)	91.13% (90.69–91.58)	84.79% (84.18–85.39)	65.59% (64.58–66.61)
Unmatched	93.82% (93.49–94.15)	87.24% (86.75–87.73)	78.34% (77.67–79.01)	54.42% (53.37–55.49)
*P*-value	<0.001	<0.001	<0.001	<0.001
Asian				
Matched	97.28% (96.75–97.82)	93.98% (93.17–94.80)	90.01% (88.91–91.12)	72.94% (70.85–75.08)
Unmatched	95.03% (94.62–95.44)	89.36% (88.74–89.98)	82.83% (82.01–83.65)	62.21% (60.86–63.59)
*P*-value	<0.001	<0.001	<0.001	<0.001

After propensity score-matching, no significant difference was found in patient survival (*P* = 0.93) (Figure 2C). Interestingly, race-matched patients continued to show longer graft survival after propensity score matching (*P* < 0.001) (Figure 2D).

### Subgroup analysis

**Figure 3:**
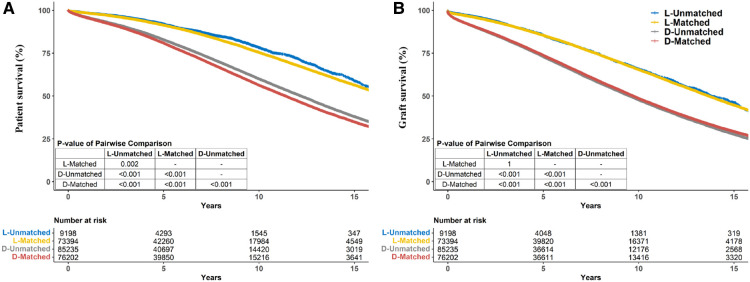
Crude kaplan–meier estimates of patient (**A**) and graft (**B**) survival for kidney transplant recipients stratified by living/deceased donor-recipient race-matched vs. unmatched groups.

**Figure 4:**
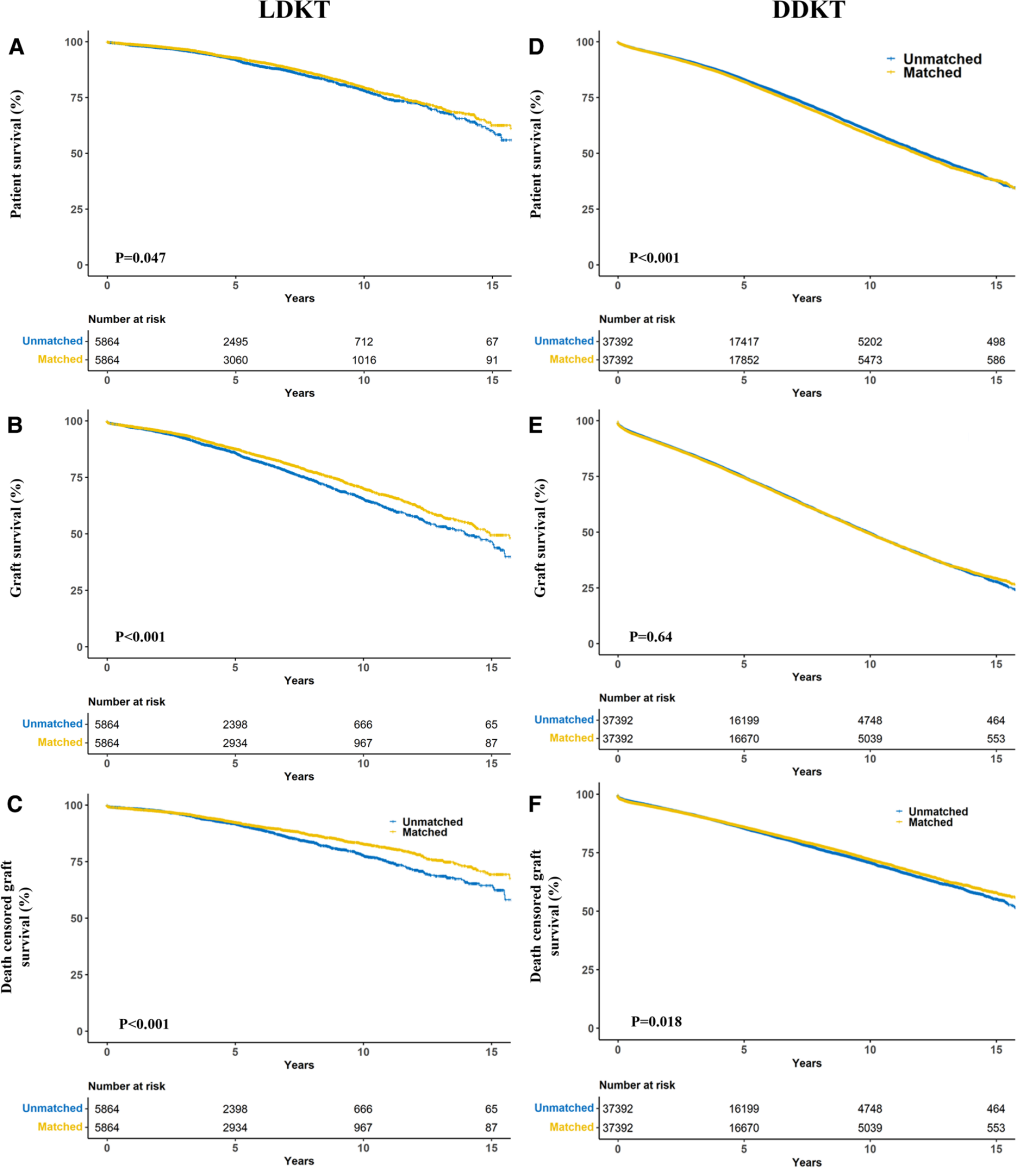
Propensity score matching kaplan–meier estimates of patient (**A,B**), graft (**C,D**) and death-censored (**E,F**) survival in living donor kidney transplant (**A–C**) and deceased donor kidney transplant (**D–F**) recipients stratified by donor-recipient race-matching.

Notably, patients who received allografts from race-unmatched living donors demonstrated longer survival than those who received allografts from race-matched living donors in the unadjusted Kaplan–Meier curves (*P* = 0.002). A similar outcome was observed in patients who received allografts from deceased donors (*P* < 0.001) (Figure 3A). Compared to race-unmatched patients, race-matched patients who received allograft from living and deceased donors experienced a 12% and 11% increase in the risk of unadjusted mortality, respectively (HR 1.12, 95% CI: 1.06–1.20, *P* < 0.001 and HR 1.11, 95% CI: 1.09–1.14, *P* < 0.001) (Table 2). After propensity score-matching, race-matched LDKT patients had a longer adjusted patient survival than unmatched control (*P* = 0.047) (Figure 4A); however, the opposite result was observed for DDKT patients (*P* < 0.001) (Figure 4D).

The survival of grafts received from race-matched deceased donors was longer than that of grafts from race-unmatched donors (*P* < 0.001) (Figure 3B), with a 3% decrease in the risk of unadjusted graft failure (HR 0.97, 95% CI: 0.96–0.99, *P* < 0.001) (Table 2). However, there was no significant difference in graft failure between race-matched and race-unmatched living donors (*P* = 1) (Figure 3B). After propensity score-matching, race-matched LDKT patients demonstrated longer adjusted graft survival than the unmatched control group (*P* < 0.001) (Figure 4B) and statistical differences still existed when deaths were censored (*P* < 0.001) (Figure 4C). However, there was no significant difference in graft survival between the race-matched and unmatched DDKT groups (*P* = 0.64) (Figure 4E). Interestingly, when deaths were censored, the survival difference between grafts received from race-matched and unmatched deceased donors was significant (*P* = 0.018) (Figure 4F).

## Discussion

We used UNOS database between 2000 and 2019 to examine the effect of donor-recipient race-matching on post-KT outcomes. In our population-based cohort analysis of 244,037 patients, we observed that donor-recipient race-matching was associated with better prognosis for crude patient and graft survival, but with higher mortality when stratified by donor type. Here is an example of Simpson's paradox (13), which are the first to identify in KT. After propensity score-matching, donor-recipient race-matching was associated with a reduced risk of overall crude graft loss but not mortality. Race-matching resulted in longer patient, graft, and death-censored graft survival in LDKT and death-censored graft survival in DDKT. However, donor-recipient race-matching was associated with an increased risk of mortality in DDKT recipients. Therefore, it may be beneficial for patients to consider donor-recipient race-matching in clinical decision-making in KT.

Low rates of patient and graft survival in African Americans receiving KT were first identified in 1977 due to racial disparities (14). Although transplant medicine has made great progress in terms of surgical techniques and immunosuppressant therapy over the past 50 years, racial disparities remain. Previous studies (12, 15–17) have shown that black patients have shorter graft survival and poorer graft function after KT. Despite inconsistent results, more recent studies (18, 19) have demonstrated that patient and graft survival among Asians and Hispanics are longer than among Caucasians. Moreover, non-African Americans experience longer patient survival than do African Americans (20, 21). Due to the disadvantages of socioeconomic status and other factors, less access to health care and specialists for African Americans may have contributed to these differences. In addition, previous researches (22, 23) have shown that the immune response is more intense in African Americans, which increases the incidence of acute rejection and chronic allograft failure. Studies have confirmed that HLA matching affects survival after KT (24). Presumably, race-matched recipients are more likely to have similar HLA genes and experience fewer acute rejection events than unmatched recipients, which may result in longer patient and graft survival. In our study, the proportion of HLA mismatches (≥3) was higher in race-unmatched recipients, supporting this hypothesis.

Donor-recipient race-matching and post-transplant outcomes have been studied for lung (8), liver (9, 25), and heart (10, 26) transplantation, and the findings showed that the outcomes are more satisfactory if donors and recipients are race matched. Allen et al. (8) performed an analysis of 11,323 primary lung transplant patients from the UNOS dataset between 1997 and 2007 and found that race-matching was associated with a reduced risk of cumulative mortality. In the risk-adjusted model, donor-recipient race-matching reduced the cumulative mortality risk (HR 0.88, 95% CI: 0.80–0.96, *P* = 0.006). Silva et al. (9) retrospectively analyzed African Americans with hepatocellular carcinoma undergoing liver transplantation from 1994 to 2015 using the OPTN/UNOS database and observed that race-matched patients had a higher median overall survival than unmatched patients (135 vs. 78 months, *P* = 0.007). In multivariate analysis, the adjusted hazard ratio for race matching was shown to be 0.66 (95% CI: 0.49–0.88, *P* = 0.004). Kanter et al. (26) conducted a single-center analysis of 169 pediatric primary heart transplants and found that donor-recipient race mismatch showed a lower 5-year graft survival rate (72.3% vs. 48.9% for matched vs. unmatched, *P* = 0.0032). Donor-recipient race mismatching was a predictor of graft failure in the multivariable Cox proportional hazards regression model (HR 2.137, 95% CI: 1.054–4.335, *P* = 0.0353). They considered HLA mismatch as a possible confounder because the incidence of HLA crossmatch was higher for black recipients than for Caucasian recipients.

LeClaire et al. (11) conducted an analysis of solid organ (including heart, lung, liver, kidney, and pancreas) transplantations from the UNOS database and reported that patient survival in KT was not influenced by race-matching, but race-unmatched African Americans experienced shorter graft survival at all time points after controlling for potential confounders, which was inconsistent with our results. In our results, the crude patient and graft survival rates in race-matching were significantly higher than those in race-unmatching. The advantages of race-matching remain in reducing graft loss after propensity score-matching, although there is no significant difference in mortality. The discrepancy between that study and our results may be explained by differences in the control of confounders, with their study analyzing based on ethnicity and ours based on donor type.

We observed that this study presented longer overall crude patient survival in the race-matched group than in the unmatched controls. However, when stratified by donor type, we found the opposite in terms of patient survival, which was longer in the race-unmatched group. This can be explained by Simpson's paradox (1): there are more DDKT recipients in the unmatched group than in the race-matched group, whereas the reverse is true for LDKT recipients, and (2) patients receiving kidneys from living donors experience dramatically longer survival than those receiving kidneys from deceased donors. Simpson's paradox is not limited to race-matching and KT outcomes and has been observed in clinical trials (27), ecological studies (28) and other fields. The results of LDKT and DDKT separately support longer patient survival for race-matched patients, but opposite outcomes are achieved when these groups are combined. After adjusting for baseline confounders, race-matching was associated longer patient survival in LDKT but shorter patient survival in DDKT. Given the variation in clinical characteristics of patients (e.g., age, recipient race and cause of ESRD), Simpson's paradox may arise without adjusting for the possibility of some factors interacting. However, the cause of this discrepancy requires further investigation.

Since the successful practice of LDKT, over 35,000 LDKT procedures are performed each year in the world (29). According to available studies (30–32), living donors have apparent advantages over deceased donors in KT, as they offer longer long-term survival, especially with young donors. However, the number of DDKT cases is far greater than that of LDKT. Surprisingly, in the present study, we found that patient survival was shorter for race-matching than for unmatching in LDKT and DDKT, which may be caused by baseline differences. However, graft survival is longer in race-matched than unmatched transplant in DDKT. In contrast to DDKT, an advantage of race-matching for patient survival after adjustment was observed in LDKT. When deaths were rigorously censored, the advantages of race-matching in graft survival persisted in both the groups. Therefore, we believe that race-matching may be a protective factor in KT, especially for graft survival. Locke et al. (3) performed a retrospective study of the UNOS between 1993 and 2006 and found a 70% reduction in graft loss among African American recipients receiving race-matched kidneys from donation-after-cardiac-death (DCD). However, the allocation of DCD organs and post-transplant care has been improved dramatically in recent years and the applicability of that study is also limited by inadequate sample size. Pisavadia et al. (12) undertook a retrospective study of primary kidney-alone transplantation in adults, using UK Transplant Registry data between 2003 and 2015 and found no statistical differences in patient and graft survival by race-matching after stratification by LDKT and DDKT. The disparities between that study and ours may be due to the different compositions of the ethnic populations in the UK and the United States. Tahir et al. (33) performed a retrospective study comparing the outcomes of Black kidney transplant recipients in the United States and the UK and found that the outcomes of kidney transplants in Blacks differed between the two countries. Therefore, we believe that the differences in racial effects between the two countries may lead to different outcomes.

Our study had several limitations. First, the study was limited by the methodology of the retrospective cohort, and we were unable to control for all potential confounders, with some important variables missing in our analysis due to the limitation of UNOS data collection. It must be noted that secondary outcomes data (e.g., rejection) in the UNOS dataset is not as complete as primary outcomes data (e.g., survival). Second, the populations of race-matched recipients are much larger for Caucasians than for African Americans, Hispanics, and Asians in KT. For example, there were 107,351 Caucasians recipients receiving matched kidneys, compared to only 20,741 African Americans, 17,927 Hispanics, and 3,581 Asians. Our conclusions may also be affected by the skewed racial distribution. Finally, the purpose of the study was not to identify the underlying mechanism associated with survival difference in donor-recipient race-matching and future studies should explore physiological mechanisms.

This analysis is a contemporary large-sample report on the prognosis of KT based on race-matching. Overall, the survival benefits for recipients improved slightly when the race of recipients was matched to that of donors. Moreover, our results identified that the confounding factors at baseline led to contorted crude conclusions in subgroups, which was reversed again to normal trends in the combined analysis due to Simpson's paradox caused by the LDKT/DDKT ratio. Given the lack of understanding of the mechanisms by which race-matching affects prognosis, this study will drive further scientific research on the immunology and genetics of race matching. However, when considering the clinical practice of KT, the impact of race-matching on patient outcomes was insufficient to affect organ transplant offers.

## Data Availability

The datasets presented in this study can be found in online repositories. The names of the repository/repositories and accession number(s) can be found below: Publicly available datasets were analyzed in this study. This data can be found here: https://optn.transplant.hrsa.gov/data/.
