# Supplementary figures and images for: Simpson's paradox and the impact of donor-recipient race-matching on outcomes post living or deceased donor kidney transplantation in the United States
Source: Front Surg. 2023 Jan 9;9:1050416. doi: 10.3389/fsurg.2022.1050416 (PMC9869683; doi:10.3389/fsurg.2022.1050416)

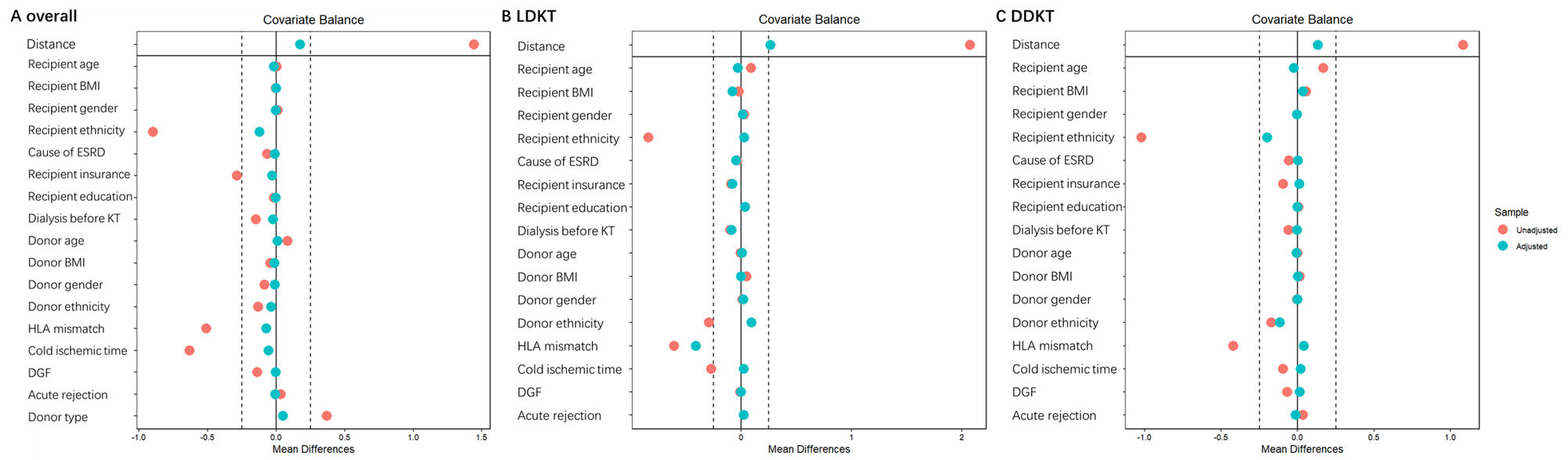

Supplement: Supplementary file 1 [file Image1.tif]
